# The Decade of Porcine Circovirus Type 2 (PCV2) in Thailand: Phylodynamic and Molecular Epidemiology

**DOI:** 10.1155/tbed/5565449

**Published:** 2025-12-06

**Authors:** Tepyuda Sritrakul, Narut Thanantong, Alongkot Boonsoongnern, Pichai Jirawattanapong, Yonlayong Woonwong, Nantana Soda, Tanyanant Kaminsonsakul, Worata Klinsawat, Porntippa Lekcharoensuk, Manakorn Sukmak

**Affiliations:** ^1^ Department of Veterinary Public Health, Faculty of Veterinary Medicine, Kasetsart University, Kamphaeng Saen, 73140, Nakhon Pathom, Thailand, ku.ac.th; ^2^ Department of Farm Resources and Production Medicine, Faculty of Veterinary Medicine, Kasetsart University, Kamphaeng Saen, 73140, Nakhon Pathom, Thailand, ku.ac.th; ^3^ Kamphaeng Saen Veterinary Diagnostic Center, Faculty of Veterinary Medicine, Kasetsart University, Kamphaeng Saen, 73140, Nakhon Pathom, Thailand, ku.ac.th; ^4^ Zoetis Thailand, Park Silom Building Convent Road Silom Subdistrict Bang Rak District, Bangkok, 10500, Thailand; ^5^ Conservation Ecology Program, School of Bioresources and Technology, King Mongkut’s University of Technology Thonburi, Bang Khun Thian Chai Thale Road Tha Kham Bang Khun Thian, Bangkok, 10150, Thailand, kmutt.ac.th; ^6^ Department of Microbiology and Immunology, Faculty of Veterinary Medicine, Kasetsart University, 50th Ngamwongwan Road Lat Yao Subdistrict Chatuchak District, Bangkok, 10900, Thailand, ku.ac.th

**Keywords:** evolution, genetic diversity, porcine circovirus type 2, recombination

## Abstract

Porcine circovirus type 2 (PCV2) is a major swine pathogen causing significant economic losses in the swine industry worldwide. Continual monitoring of genetic and antigenic diversity is essential for the early detection of emerging variants. This study investigates the evolutionary dynamics and genetic variation of PCV2 circulating in Thailand and across Asia from 2007 to 2024, using integrated phylogenetic and phylodynamic analyses. Analysis of 2739 PCV2 genomes, after excluding 0.99% recombinant strains, revealed four main groups circulating in Asia, with PCV2d as the predominant genotype. PCV2d has diversified into several distinct clades, including a recently identified variant with the ^133^HDAM^136^ amino acid motif, which likely originated from earlier variants, the ^133^ANAL^136^ and ^133^ATAL^136^ motifs. Recombination analysis detected intragenotypic recombination events within PCV2d strains circulating in Thailand, emphasizing the role of genetic recombination in driving the evolutionary changes of the virus. Phylodynamic analysis demonstrated significant fluctuations in the virus population size, correlating with changes in genotype dominance over time. Selective pressure analysis identified positively selected sites in the capsid protein (codons 63, 131, 134, 169, and 190), indicating ongoing adaptation under host immune pressures. Structural modeling and epitope analyses revealed mutations affecting antigenic sites and immune recognition, suggesting concerns for vaccine efficacy. This integrated approach enhances our understanding of PCV2 evolution, informing strategies for effective vaccine development and disease control.

## 1. Introduction

Porcine circovirus type 2 (PCV2) is a major pig pathogen, causing significant economic losses to the global swine industry. It is the causative agent of porcine circovirus‐associated diseases (PCVAD), which are clinically associated with respiratory, enteric, reproductive and systemic signs, and increased mortality particularly in weaned piglets [1]. PCV2 belongs to the genus *Circovirus* in the family *Circoviridae*. It is a small, nonenveloped virus with a circular, single‐stranded DNA genome [2] of ~1.7 kb [3]. The genome contains two major open reading frames: ORF1, which encodes the replicase protein (Rep), and ORF2, which encodes the capsid protein (Cap) [4, 5]. The capsid protein facilitates viral entry into host cells and contains the main antigenic regions targeted by neutralizing antibodies and cell‐mediated immunity. PCV2 has a relatively high evolutionary rate, estimated at ~1.2 × 10^−3^ substitutions/site/year [6], which is comparable to single‐stranded RNA viruses. Among the viral genes, ORF2 evolves faster than the full genome. Due to its high variability, ORF2 is commonly used for molecular epidemiology and genotyping studies [7, 8].

Three genotypes of PCV2, including PCV2a, PCV2b, and PCV2d, have been widely associated with clinical diseases [9, 10]. PCV2a was the first identified and predominated genotype before the introduction of vaccines. In the early 2000s, PCV2a was replaced by the emergence of PCV2b, which was subsequently dominated by PCV2d. Currently, PCV2d is the dominant genotype worldwide [9, 11]. Other genotypes such as PCV2c, PCV2e, and PCV2f are less prevalent and their clinical significance remains unclear [12]. PCV2i is the most recently reported genotype; however, further studies are needed to clarify its epidemiology and pathogenic potential [11]. In Thailand, PCV2 was detected since 1993 with retrospective study. The first clinical outbreak was reported in 1998 [13, 14]. Genotype characterization of PCV2 in Thailand identified PCV2b as the initial dominant genotype during 2004–2011 [13]. PCV2d was classified by the signature motifs and topology of ORF2 in Thailand since 2009 and clearly shifted from genotype B to genotype D during 2013–2014 [10]. The insight into genetic diversity, evolution, and phylodynamic of PCV2 in Thailand and Southeast Asia and how it is related to the global PCV2 is epidemiologically important. However, this information is quite limited to a few studies [15–18].

This study aims to update the current understanding of the evolutionary dynamics and genetic variation of PCV2 circulating in Thailand and across Asia from 2007 to 2024 using integrated phylogenetic and phylodynamic analyses. By analyzing both full‐genome sequences and the capsid gene, this study provides an overview of genotype distribution, recombination patterns, and population changes over time. Comparative analysis of genetic variation and capsid protein structure will enhance our understanding of PCV2 evolution and support the development of more effective cross‐protective vaccines for swine populations.

## 2. Materials and Methods

### 2.1. Sample Collection/DNA Extraction/PCR/Sequencing

During 2022–2024, the 34 PCV2 clinical samples, for example, lymph‐node, serum, whole‐blood were collected from suspected PCV2‐infected pigs that underwent necropsy performed by veterinarians at Kamphaeng Saen Veterinary Diagnostic Center (KVDC), Faculty of Veterinary Medicine, Kasetsart University, Thailand. The DNA extraction was performed using FavorPrep viral nucleic acid extraction kit (Favorgen, Taiwan), following the manufacturer’s protocol. For PCV2 detection, Real‐time PCR was performed. The primers and probes were used and adjusted according to the previously described [19]. The whole genome sequence was amplified by PCR using two sets of overlapping primers [20]. The obtained PCR products were purified and sent for Sanger sequencing at First Base Laboratory, Shah Alam, Malaysia. A total of 34 representative PCV2 sequences, selected from different time periods and farms, were obtained for further analysis. This study was approved by the Institutional Animal Care and Use Committee (IACUC) (ACKU64‐VET‐063 and ACKU67‐VET‐081) of Kasetsart University, Thailand.

### 2.2. Dataset Preparation and Recombinant Analysis

The complete genome sequences of PCV2 reported in Asia during 2007–2024 were retrieved from the NCBI Virus database (National Center for Biotechnology Information, https://www.ncbi.nlm.nih.gov/labs/virus/vssi/#/), accessed on October 14, 2024. Likewise, metadata that contained collection year and country were prepared. The total of 3254 PCV2 sequences including 34 sequences from this study (GenBank accession numbers are provided in the Supporting Information 1) were aligned using MAFFT (Multiple sequence alignment software V.7) [21, 22] web server. To reduce redundancy in the alignment, sequences were filtered to retain only representative sequences with nucleotide differences at any position (threshold = 0). Sequence filtering was performed using a custom script based on Biopython [23]. This ensures that sequences differing by even a single nucleotide were included while removing identical duplicates. The sequences were filtered to retain 2739 unique representatives, which were subsequently used to screened for recombination prior to downstream analyses.

The recombination analysis was performed using RDP v4.101 [24] and Simplot v3.5.1. The recombination events were evaluated using RDP [25], GENECOV [26], Bootscan [27], Chimera [28], Maxchi [29], SiSscan [30], and 3Seq [31] methods available in RDP v4.101. The recombination was confirmed when the *p*‐value was set to 0.05 and required agreement from at least five out of seven methods. Simplot v.3.5.1 analysis was performed with the following parameters: a window size of 200 bp, a step size of 20 bp, 1000 replicates, and the Kimura 2‐parameter distance model. Putative recombinant genomes identified by either method were inspected and excluded from phylogenetic, selection and trait analyses to prevent artefactual topologies and false‐positive selection signals.

### 2.3. Phylogenetic Analysis of PCV2 Circulating in Thailand and Asia During 2007–2024

The nonrecombinant subset (*n* = 2712) derived from the 2739 representative genomes was used to construct a maximum likelihood (ML) tree using IQ‐TREE v.2 [32]. The best‐fit nucleotide substitution model was determined using ModelFinder implemented in IQ‐TREE v2, and the general time reversible (GTR) + G model was selected based on the Bayesian Information Criterion (BIC). Branch supports were assessed with 1000 ultrafast bootstrap (UFBoot2) replicates to evaluate tree reliability [33]. The resulting phylogenetic tree file was subsequently combined with metadata, including the year of sample collection and the country of origin, to generate a tree visualization using the Interactive Tree of Life (iTOL) v.5 software [34] to illustrate the geographic and temporal distribution of PCV2 lineages across Asia. PCV2 strains and countries of origin used in the phylogenetic analysis are provided in Supporting Information 2.

### 2.4. Discrete Trait Inference and Counting Introductions Into Thailand

To quantify introductions of PCV2 into Thailand, the Asia‐wide complete‐genome dataset was analyzed. Sequences were aligned, and a ML tree (IQ‐TREE, GTR + G) was generated. We excluded putative recombinants (curation; RDP/SimPlot) to avoid recombination‐driven distortions in topology/branch lengths and biased geographic transition counts. The ML tree was then refined with Augur (Nextstrain) [35] under a strict molecular clock (fixed rate = 1.2 × 10^−3^ substitutions/site/year), marginal date inference, and a skyline coalescent prior and exported as a time‐resolved tree to Auspice 2.61.2 [35]. Discrete ancestral states at the country level were reconstructed with augur traits, retaining node‐level posterior probabilities. An introduction into Thailand was defined as a branch showing a transition from a non‐Thailand parent to a Thailand child, and events were enumerated from the exported JSON, retaining only those with child‐state posterior ≥0.60 in the primary analysis (with ≥0.70 as a sensitivity check). Because sampling is uneven and we applied a conservative threshold, counts are interpreted as a lower‐bound estimate. This approach follows published study that estimates viral introductions by mapping discrete geographic state changes on time‐scaled phylogenetic trees [36].

### 2.5. Phylodynamic of PCV2 Circulated in Thailand During 2007–2024

Since the *rep* gene has limited data and may not effectively explain the evolution and adaptation of the virus as well as the *cap* gene; thus, the *cap* gene was used to construct the phylodynamic analysis of PCV2 circulating in Thailand during 2007–2024. The data set was established using 286 sequences of complete (702 bp) or partial *cap* gene of PCV2 including 34 sequences in this study and 252 available sequences from NCBI Virus database (https://www.ncbi.nlm.nih.gov/labs/virus/vssi/#/, accessed on October 14, 2024). When sequences had different lengths, N nucleotides were inserted to standardize the sequence lengths. The phylogenetic tree was generated using a pipeline adapted from the Zika virus workflow available on the nextstrain.org website [35]. This process utilized Augur [35], MAFFT [21, 22], and IQ‐TREE software using ML method with GTR model [37]. The ML phylogenetic tree was then refined using TreeTime [38] to generate the time‐resolved phylogenetic tree. The data of collection year of each PCV2 was supplemented as metadata (Supporting Information 1) then associated with TreeTime using Augur. The results were exported via Augur and the phylodynamic tree was visualized in Auspice 2.61.2 [35]. The phylodynamic tree presented the information on colors, nodes, and collection years of samples. Following the time‐scaled phylogenetic reconstruction, the capsid gene sequences were further analyzed to identify codon‐level selection signals, which may underlie observed patterns of viral adaptation.

### 2.6. Selective Pressure Analysis

The Cap protein was used as the representative to verify the pervasive selective pressure of PCV2 circulated in Thailand during 2007–2024. All nucleotide sequences were aligned and examined for recombination prior to selection analysis. The single‐likelihood ancestor counting (SLAC) [39], fast unconstrained Bayesian approximation (FUBAR) [40], fixed‐effects likelihood (FEL) [39], and mixed effects model of evolution (MEME) [40] were applied to estimate the selective pressure based on the ratios between nonsynonymous and synonymous substitution rates (dN/dS) using Datamonkey web server [41].

### 2.7. Demographic Changes Over Time (Bayesian Skyride Plot)

Bayesian skyline plot (BSP; [42]) and Bayesian skyride plots with Gaussian Markov random fields (time‐aware smoothing of GMRF Skyride; [43]) of the demographic changes of the virus over time were reconstructed with *cap* gene (ORF2) and strict molecular clock in BEAST v1.10.4 [44]. Due to limited rate variation among PCV2 variants, the strict clock model was selected over the uncorrelated lognormal relaxed clock model based on model comparison using log Bayes factors (BF > 3) [45, 46]. When the skyline coalescent tree prior was compared against skyride coalescent tree prior using the path sampling method [45], the BF for the marginal log likelihood of the model was significant (BF > 3). Therefore, skyride tree prior model was selected to estimate a temporally smoothed trajectory of PCV effective population size. Similarly, the path sampling approach [45] provided support for the skyride coalescent tree prior over the coalescent skyline prior (BF > 3). The skyride’s Gaussian Markov random field smoothing allows effective population size to change continuously over time, reducing noise from the skyline’s abrupt piecewise‐constant changes and providing a more accurate evolutionary inference for the PCV2 population dynamics.

During the inferred phylodynamic processes, the y‐axis represented effective size (in natural log scale) of PCV2 population in Thailand domestic pig (*N*_e_T, where *N*_e_ is the effective population size and T is the generation time [43]) and the x‐axis showed the timeline from 1991 (the root of PCV2 in Thailand) to 2024. The best‐fitted nucleotide substitution model was GTR + G (gamma) model with empirical base frequencies. Normal distribution prior for the clock rate (mean = 7.38 × 10^−4^, standard deviation = 0.5) was set to reflect high evolutionary rates of PCV2 and encompass the reported range of PCV2 nucleotide substitutions per site per year [6]. Tuning and weight of some operators were adjusted to allow proper move to explore parameter space and ensure proper Markov Chain Monte Carlo (MCMC) mixing and convergence. Weight of clock rate scale factor increased from the default 3.0–10.0 and similarly and weights of tree topology operators including tree wide Exchange and Wilson Balding increased from 3.0 to 30.0. Two independent Bayesian MCMC analyses were carried out as follows: a chain of 2 × 10^8^ generations, sampling every 2 × 10^4^ (10,000 samples), and the first 20% of the samples were discarded as burn‐ins to provide reliable approximations of the posterior probability densities of each parameter. Tracer v1.7.2 [47] was used to ensure convergence of the MCMC runs and independent samples from the parameter space supported by effective sample sizes (ESSs) of all posterior parameters > 200.

### 2.8. Structural Modeling of PCV2 Capsid Protein

The PCV2 isolated 374TH2023 and 10131TH2024 were used as the representative of ^133^HDAM^136^ and ^133^ANAL^136^ clades, respectively [17]. The amino acid sequences (233 aa) were used to generate models. Monomer and trimer of PCV2 capsid structures were predicted by AlphaFold server [48] (https://alphafoldserver.com). The predicted structures were visualized and compared to PCV2 template structure from Protein Data Bank (PDB ID: 3R0R) using ChimeraX 1.8 software [49]. Quality of the predicted structures was evaluated using Ramachandran plot analysis.

### 2.9. T‐Cell Epitope Prediction

T‐cell epitope prediction was carried out using the amino acid sequence translated from ORF2 genes. Twenty proteins were selected from PCV2a, PCV2b, and three clades of PCV2d as representative examples for prediction. The Immune Epitope Database (IEDB) analysis tool, a freely available online resource for epitope analysis, was mainly used for prediction (https://www.iedb.org). We applied the MHC binding to predicted peptide binding to specific MHC molecule. For CD8 T cell epitope, the NetMHCpan 4.1 EL and NetMHCpan 4.1 BA methods [50, 51] were used to predict the ability of peptide to bind to MHC molecule and processed for T cell recognition. Three SLA class I alleles (SLA‐1 ^∗^08:01, SLA‐2 ^∗^02:01, SLA‐3 ^∗^04:01) were selected based on their high frequently occurring alleles in commercial pig population in Thailand [52]. Since swine MHC class II alleles were unavailable in the prediction tool, CD4 T‐cell epitope prediction was performed using the human alleles DRB1 ^∗^04:01, DQB1 ^∗^02:01 and DQA1 ^∗^02:01. The predicted peptides with high prediction score and IC50 value of <500 nM were selected as strong T‐cell epitopes.

### 2.10. Linear and Discontinuous B‐Cell Epitope Prediction

The capsid protein sequences and predicted structures of PCV2d were used to predict linear and discontinuous epitopes. Linear B‐cell epitope prediction was performed using the Antigenic Sequence Properties analysis tool available in IEDB. This tool incorporates multiple prediction methods, including Bepipred 2.0 epitope prediction [53], Chou and Fasman beta‐turn prediction [54], Emini surface accessibility prediction [55], Karplus and Schulz flexibility prediction [56], Kolaskar and Tongaonkar antigenicity prediction [57], and Parker hydrophilicity prediction [58]. All these methods were applied for comprehensive B cell epitope prediction. High‐scoring regions were considered potential linear B‐cell epitopes. The modeled 3D capsid structures were used for discontinuous B‐cell epitope prediction using ElliPro, an antibody epitope prediction tool [59]. ElliPro identifies both linear and discontinuous B‐cell epitopes based on the protein’s 3D structure. The prediction parameters included a minimum score threshold of 0.5 and a maximum distance of 6 Å. The predicted discontinuous epitopes were analyzed, and residues with a high protrusion index were considered potential B‐cell epitopes. The structures were visualized and displayed with Pymol version 3.1.3.

## 3. Results

### 3.1. Recombination in PCV2 Genomes and Intragenotypic Recombination in Thai Isolates

**Figure 1:**
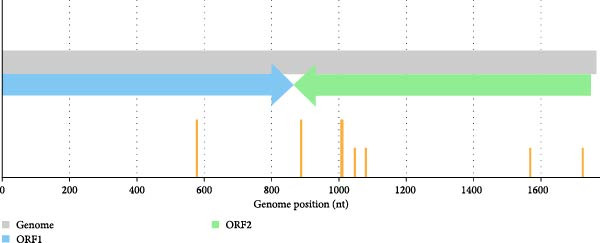
Visualization of the top 10 recombination breakpoint hotspots in PCV2 genomes. The gray bar represents the full PCV2 genome (1767 nt). The blue and green arrows indicate the positions and directions of ORF1 and ORF2, respectively. Orange bars represent the top 10 most frequently identified breakpoint positions, as determined from 27 recombinant sequences. Breakpoints are concentrated in the region between ORF1 and ORF2, suggesting a possible recombination hotspot in the intergenic junction.

Out of 2739 representative PCV2 sequences, 27 recombinant strains were identified, accounting for ~0.99% of the dataset. These recombinant events were detected using multiple recombination detection methods, and each recombinant was supported by statistically significant *p*‐values (Supporting Information 3). In some cases, the parental sequences were initially labeled in bracket (e.g., KR704908) indicating probable parents based on sequence similarity, though without conclusive assignment. Breakpoints were distributed across the genome, with some regions—such as between positions ~1000–1200 showing higher frequency, possibly indicating recombination hotspots (Figure 1). Several parental lineages, such as OQ785638 and MK426836, appeared recurrently across events.

**Figure 2:**
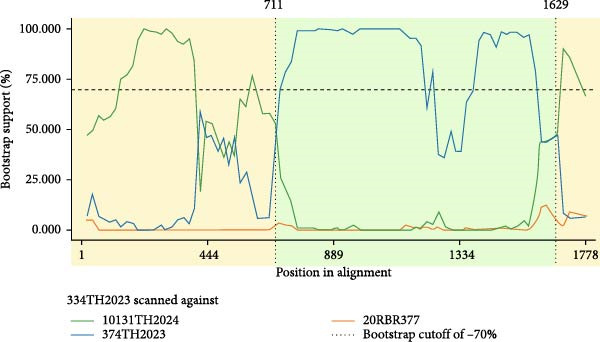
The bootscanning analysis revealed the recombination event of 334TH2023. The recombination breakpoints were identified at nucleotide positions 711 and 1629. Yellow and green shades highlighted the recombination regions.

However, analysis of 34 PCV2 isolated in Thailand during 2022–2024 showed only one event of genetic recombination. All 7 methods (RDP, GENECONV, BootScan, MaxChi, Chimaera, SiScan, and 3Seq) indicated that 334TH2023 was a recombinant strain with high degree of statistical support (average *p*‐value, 2.7 × 10^−3^). The PCV2 isolates 374TH2023 and 10131TH2024 were identified as major and minor parental sequences, respectively. Since, all of isolates was PCV2d, these results indicated intragenotypic recombination. The Simplot v3.5.1 was used to confirm the recombination event. Here, we used 20RBR377 (OL677614), which confirmed as PCV2b as an outgroup for comparison. The recombination breakpoints were identified at nucleotide position 711 and 1629 (Figure 2). The PCV2 isolate 374TH2023 was clustered in ^133^HDAM^136^ clade while 10131TH2024 was clustered in ^133^ANAL^136^ clade. However, isolate 334TH2023 was clustered in ^133^ANAL^136^ clade.

### 3.2. Phylogenetic Analysis of PCV2 Circulating in Thailand and Asia During 2007–2024

**Figure 3:**
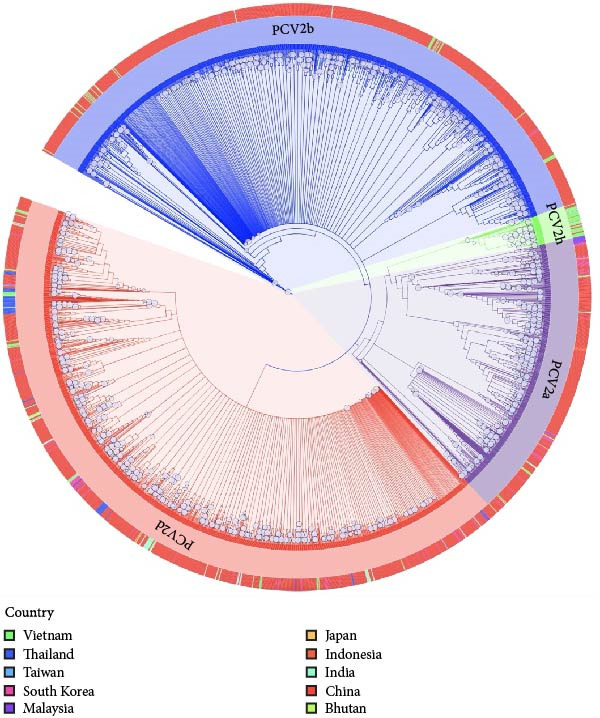
The maximum likelihood phylogenetic tree of 2712 complete genomes sequences (1766–1788 bp) of PCV2 sequences circulating in Asia from 2007 to 2024. Recombinant sequences identified through RDP4 were excluded prior to tree reconstruction. The colored strips around the outer circle represent sequences originating from different countries, as indicated by the labels in the legend. The colors of the internal branches denote the grouping of PCV2 genotypes: blue branches represent PCV2b, purple branches represent PCV2a, green branches represent PCV2h, and red branches represent PCV2d. Bootstrap support values greater than 70 are indicated by gray circles at the corresponding nodes.

The phylogenetic reconstruction using 2712 complete genome sequences of PCV2 collected from various Asian countries between 2007 and 2024 revealed four genotypes, namely PCV2a, PCV2b, PCV2d, and PCV2h (Figure 3). Recombinant sequences identified through RDP4 were excluded prior to the analysis to avoid potential bias in tree topology. The resulting tree showed three major clusters corresponding to PCV2a, PCV2b, and PCV2d, consistent with previously reported global classifications. The PCV2d lineage represented the dominant genotype circulating widely across Asian regions, including Thailand, whereas PCV2b and PCV2a were observed in smaller clusters. A minor lineage related to PCV2a, corresponding to PCV2h, was also observed but positioned closely to the PCV2a clade rather than forming a distinct group. Thai isolates collected during 2022–2024 were all classified within the PCV2d clade, indicating that this genotype remains endemic in Thailand. Overall, the updated phylogenetic tree reflects the genetic diversity and regional distribution of PCV2 in Asia.

Geographic introductions of PCV2 into Thailand Introductions were assessed on the Asia‐wide whole‐genome time‐scaled phylogeny after excluding putative recombinants. Discrete country‐level states were reconstructed on this tree, and transitions into Thailand were enumerated using a conservative posterior threshold (≥0.60). Among the Thai complete genomes represented in this dataset (*n* = 59; including 34 generated in this study), we inferred at least 12 independent introductions during 2014–2021. Given the Asia‐only sampling frame and the exclusion of ambiguous nodes, these counts represent a minimum (lower bound) within our dataset; accordingly, we do not attribute specific source locations. These counts are not directly comparable to the ORF2 phylodynamic analysis (*n* = 286 Thai sequences). An interactive, time‐scaled tree with country annotations is provided as dataset in Supporting Informations 4 and 5.

### 3.3. Phylodynamic Analysis Demonstrating Two Major Clades of PCV2d Currently Present in Thailand

**Figure 4:**
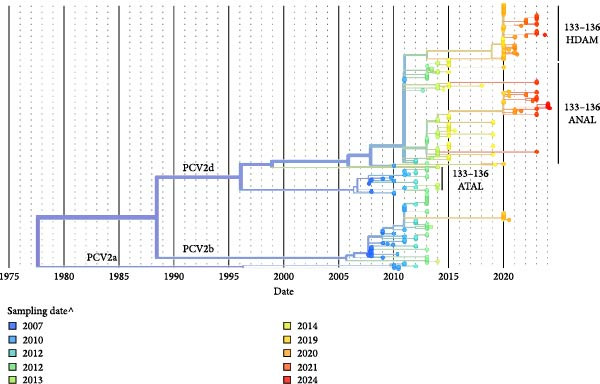
The time‐scaled phylogenetic tree of ORF2 (702 bp) of 286 PCV2 sequence circulated in Thailand during 2007–2024. This time‐scaled tree depicts the genetic relationships of PCV2 strains sampled over time, grouped into PCV2a, PCV2b, and PCV2d. PCV2d is further divided into three clades based on amino acids 133–136: ATAL, ANAL, and HDAM. The colors of the branches and nodes represent the sampling years, as indicated in the legend.

The time‐scaled ORF2 phylogeny revealed that three genotypes of PCV2 circulated in Thailand were included PCV2a, PCV2b, and PCV2d during 2007–2024 (Figure 4). PCV2d has been the predominant genotype since 2014, while PCV2b continues to be detected with a low incidence to this day. The PCV2d can be classified into three major clades based on amino acid composition at positions 133–136 of ORF2, that is, ^133^ATAL^136^, ^133^ANAL^136^, and ^133^HDAM^136^.

The final descendant of ^133^ATAL^136^ clade was observed in 2014. The ^133^ANAL^136^ clade has been continuously detected from 2009 to the present. The ^133^HDAM^136^ clade has been identified as a novel PCV2d clade in Thailand and is predicted to have started evolving around 2011, based on phylogenetic tree analysis. This result indicated that the ^133^ANAL^136^ and ^133^HDAM^136^ clades are the two major PCV2d clades currently circulating in Thailand.

### 3.4. Positively Selected Codon of the *Cap* Gene

The positive selection of the *cap* gene was analyzed, and the codon sites listed in the table below (Table 1) were identified through detection by at least two different methods (for SLAC, FEL, and MEME, *p*‐value < 0.1, and posterior probability of FUBAR > 0.90). Prior to the analysis, one recombinant sequence (334TH2023) was excluded to avoid bias in site‐by‐site estimation of selective pressure. Five individual codons, including 63, 131, 134, 169, and 190, were confirmed under positive selection. The codon 190 was consistently detected across all four methods, suggesting a strong pervasive signal of positive selection. The codons 63, 131, 134, and 169 were detected by at least two methods, indicating possible episodic diversifying selection acting on these sites.

**Table 1:** Selection pressure analysis results of capsid gene of PCV2.

Codon	SLAC		FEL		FUBAR		MEME	
	dN–dS	*p*‐Value	*α* = *β*	*p*‐Value	*β*−*α*	Post. Pro	*β*+	*p*‐Value
63	—	—	1.720	0.099	3.571	0.960	—	—
131	—	—	1.060	0.032	3.202	0.958	13.169	0.046
134	—	—	—	—	5.689	0.964	105.740	0.009
169	—	—	—	—	6.320	0.913	156.464	0.009
190	3.972	0.041	1.790	0.022	6.590	0.995	58.950	0.025

*Note:* Positive selection pressure was considered acceptable when the *p*‐value < 0.1 in SLAC, FEL, and MEME, and the posterior probability > 0.90 in FUBAR. Sites under positive selection were confirmed detection by at least two methods. “—” indicates nonsignificant results.

### 3.5. Population Dynamic of PCV2 in Thailand From 2007 to 2024

**Figure 5:**
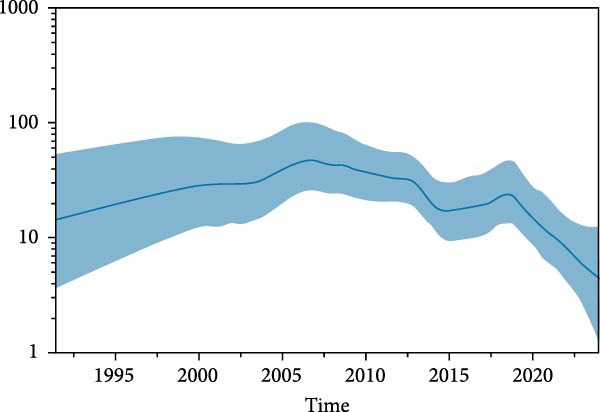
Bayesian skyride plots for complete capsid gene of PCV2 population in Thailand. The x‐axis showed the years before 2024. The y‐axis showed effective viral population size. The thicker bold line represents the median estimate of the effective number of infections over time, while the thinner blue lines depict the 95% HPD interval, showing the upper and lower bounds.

The population dynamic of PCV2 was estimated by Bayesian Skyride plot using *cap* gene. The effective population size of PCV2 gradually increased until 2007, followed by a slow decline until 2013. From 2013 to 2015, there appears to be a significant decrease, followed by a slow increase until 2019, and then a dramatic decline continuing to the present (Figure 5).

### 3.6. Surface‐Exposed Amino Acid Variations Identified in PCV2 Capsid Protein Structures

The structure models of 374TH2023 and 10131TH2024 were predicted by AlphaFold server and compared with 3R0R (2.3 Å structure of porcine circovirus 2, used as template structure) [60]. The predicted structures are similar to the template, and both predicted capsid proteins exhibited 0.8 pTM (predicted template modeling) score. Additionally, the predicted models were analyzed using a Ramachandran plot. The results showed that 89.6% and 90.0% of amino acid residues were located in the most favored regions, while 10.4% and 10.0% were in the additionally allowed regions for the 374TH2023 and 101TH312024 capsid structures (Supporting Information 6).

Structural comparison of the capsid proteins from strains 374TH2023 and 10131TH2024 revealed amino acid substitutions at positions 133, 134, 136, and 169. These mutated residues are located on the exterior surface of the capsid (Figure 6), where changes in amino acid and side chain orientation can affect the capsid structure and its interactions with other molecules.

Figure 6Trimeric structures of the 374TH2023 (A) and 10131TH2024 (B) capsid proteins. The four amino acids highlighted in the red box represent the differences in the amino acids on the capsid surfaces.

**Figure:**
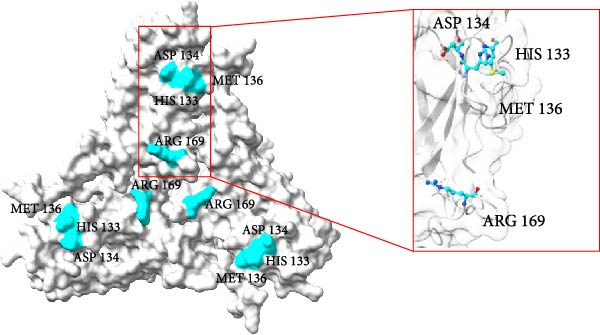
(A)

**Figure:**
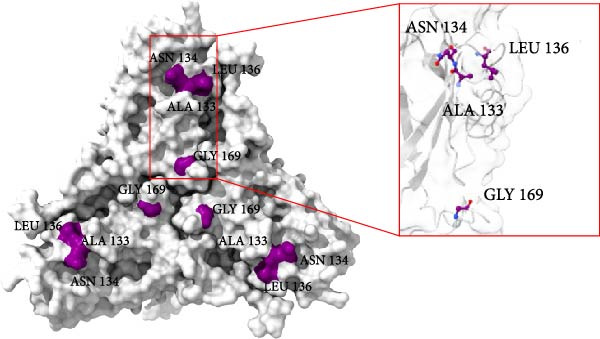
(B)

### 3.7. Predicted T‐ and B‐Cell Epitopes of PCV2 Capsid Proteins

**Figure 7:**
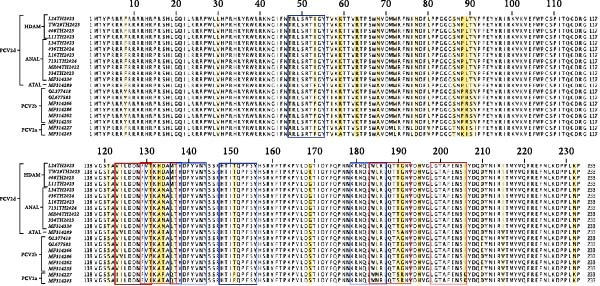
T‐cell epitopes of the PCV2 capsid protein. The figure illustrates the sequence alignment of 20 representative capsid proteins from PCV2a, PCV2b, and three clades of PCV2d. Yellow highlights indicate conserved mutation locations, while blue rectangles mark the positions of MHC class I T‐cell epitopes, and red rectangles indicate the positions of MHC class II T‐cell epitopes.

The peptide sequences of 20 capsid proteins were analyzed to identify locations suitable for T‐cell epitopes using prediction tools available on the IEDB data analysis platform (Figure 7). The peptide with the highest predicted binding score to the MHC class I allele SLA‐1 ^∗^08:01 was ^136^MTYDPYVNY^144^, while ^179^KRNQLWLRL^187^ was predicted to bind with the SLA‐3 ^∗^04:01 allele. For MHC class II binding, the peptide with the highest predicted score was ^198^LGTAFENSI^206^, which binds to the HLA‐DQA1 ^∗^02:01/DQB1 ^∗^02:01 allele. Additionally, core peptides ^123^VILDDNFVT^131^ and ^184^WLRLQTSAN^192^ were predicted to bind with the HLA‐DRB1 ^∗^04:01 allele. Most T‐cell epitopes located in regions of mutated peptides. The ^136^MTYDPYVNY^144^ peptide of the capsid protein from PCV2d (^133^HDAM^136^) exhibited the highest predicted binding score and a favorable IC50 value, while ^136^QTYDPYVNY^144^ from PCV2a and ^136^LTYDPYVNY^144^ from PCV2b, PCV2d (^133^ANAL^136^), and PCV2d (^133^ATAL^136^) showed lower predicted scores and high IC50 value, indicating reduced binding affinity to MHC class I. Similarly, mutations also influenced the ability of peptides to bind to MHC class II. The ^184^WLRLQTSAN^192^ core peptide from PCV2d (^133^ANAL^136^) had the highest predicted binding score and was derived from the mutated sequences ^184^WMRLQTSRN^192^ in PCV2a and ^184^WLRLQTAGN^192^ in PCV2b. Conversely, the lowest binding affinity was observed in the ^184^WLRLQTTGN^192^ peptide from PCV2d (^133^HDAM^136^) and PCV2d (^133^ANAL^136^) compared to other clades at the same peptide position.

Linear B‐cell epitopes were predicted based on hydrophilicity, flexibility, accessibility, turns, exposed surface, polarity, and antigenic propensity of polypeptide chains. Residues with scores above the threshold in each method indicated the locations of the predicted epitopes. The prediction focused on the ^133^HDAM^136^ and ^133^ANAL^136^ clades of PCV2d, as these clades were recently identified. The prediction patterns for ^133^HDAM^136^ (Figure 8A) and ^133^ANAL^136^ (Figure 8B) clades were nearly identical, with slight differences in scores at mutation sites. Discontinuous B‐cell epitopes were also predicted using Ellipro, which analyzes protein 3D structures. Eleven and 10 discontinuous B‐cell epitopes were predicted for the ^133^ANAL^136^ and ^133^HDAM^136^ clades, respectively (Table 2). Ten of these epitopes located in similar regions across both clades (Figure 9), with minor variations in prediction scores, likely due to differences in structural analysis. Notable differences were observed in two epitope regions. First, the region comprising residues D126, D168, G169, T170, I171, and D172 in the ^133^ANAL^136^ clade was predicted with a lower epitope score (0.825) compared to the corresponding region in the ^133^HDAM^136^ clade (D168, T170, I171) with a score of 0.937. Additionally, no corresponding ^133^HDAM^136^ epitope was found at the same position as epitope cluster 9 in the ^133^ANAL^136^ clade. Some predicted epitopes, specifically clusters 4, 7, 10, and 11, were not exposed on the capsid surface (Figure 9B).

Figure 8Linear B cell epitopes of PCV2 capsid protein. Peptide sequences of PCV2 capsid protein were analyzed by Bepipred linear epitope prediction (A1, B1), Chou and Fasman beta‐turn (A2, B2), Emini surface accessibilities (A3, B3), Karplus and Schulz flexibility (A4, B4), Kolaskar and Tongaonkar antigenicity (A5, B5), and Parker hydrophilicity (A6, B6) prediction methods. The figure presents the prediction result comparing between 133HDAM136 (A) and 133ANAL136 (B) clades. The X‐axis represents the peptide position, while the Y‐axis denotes the predicted score. Residues with scores exceeding the threshold are highlighted in yellow, indicating potential epitope regions.

**Figure:**
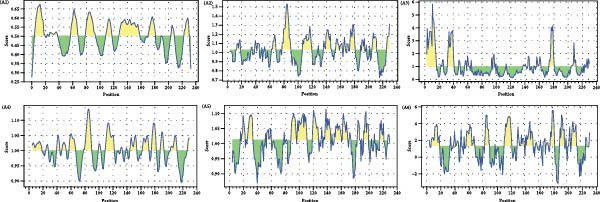
(A)

**Figure:**
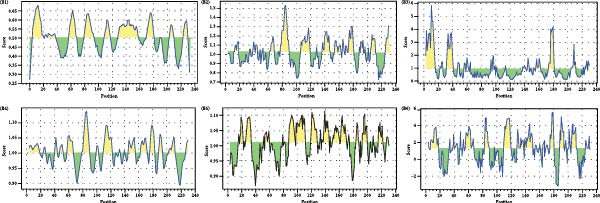
(B)

Figure 9Illustrations of the predicted discontinuous B‐cell epitopes of the PCV2d capsid protein. Eleven predicted epitope regions (blue spheres) are shown on the capsid monomer (A), while the corresponding dark blue regions are displayed on the capsid trimer (gray) in a particle model (B).

**Figure:**
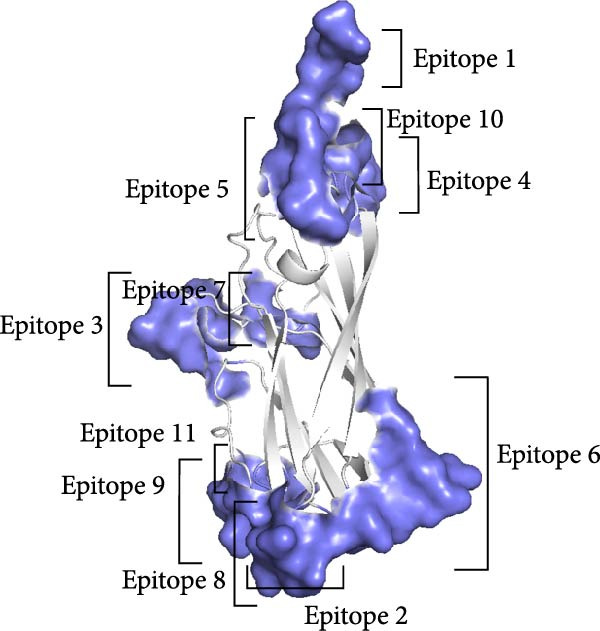
(A)

**Figure:**
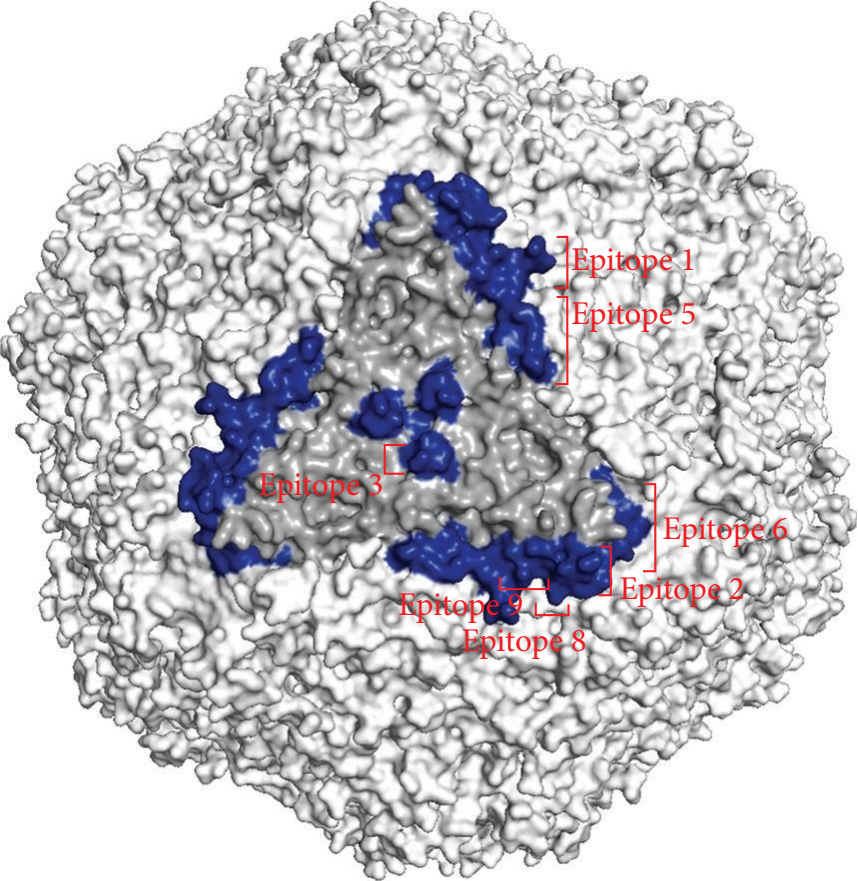
(B)

**Table 2:** The predicted discontinuous B cell epitopes of PCV2d (^133^HDAM^136^ and ^133^ANAL^136^ clades).

Epitope cluster	Number of residues, epitope score		Residues^a^
	ANAL clade	HDAM clade	
1	4, 0.987	4, 0.987	D228, P229, P230, L231
2	4, 0.862	4, 0.866	T60, T61, V62, R63
3	6, 0.825	3, 0.937	(D126), D168, (G169), T170, I171, (D172)
4	3, 0.798	3, 0.796	N42, G43, I44
5	9, 0.785	9, 0.785	P81, P82, G83, G84, G85, S86, N87, P88, L89
6	14, 0.78	14, 0.783	C108, S109, P110, I111, T112, Q113, G114, D115, P153, F154, S155, S205, I206, Y207
7	3, 0.74	3, 0.742	Q175, P176, N177
8	3, 0.709	3, 0.709	T64, P65, S66
9	5, 0.649	—	A135, L136, T137, Y138, P140
10	4, 0.647	4, 0.645	V91, P92, F224, N225
11	3, 0.604	3, 0.602	D139, V142, N143

^a^Residues that were not included in the predicted epitopes (in the ^133^HDAM^136^ clade) are indicated in parentheses.

## 4. Discussion

Recombination analysis of 2739 PCV2 sequences, including Thai isolates, clarifies how the virus evolves. Recombination was detected in 0.99% of the sequences, with breakpoints predominantly clustered between ORF1 and ORF2. This region corresponds to previously reported global recombination hotspots located at intergenic junctions [61, 62]. Although this proportion is lower than the ~33% reported [61], both analyses identified similar breakpoint hotspots and recurrent intragenotypic PCV2d recombination. The lower frequency observed in this study likely reflects the dominance of a single genotype (PCV2d) across Asia during 2007–2024 and the stringent detection criteria requiring concordance across multiple methods. The concentrated recombination breakpoint located at genome positions 1000–1200 within ORF2, which encodes for the capsid protein, may affect immunogenic epitopes. Intergenotypic PCV2b and PCV2d recombinants, such as 19NPT29, highlight the adaptability of PCV2 genomes, with recombination breakpoints in ORF1 and ORF2 [17]. This study demonstrates intragenotypic recombination within PCV2d, as seen in strain 334TH2023 derived from the ^133^HDAM^136^ and ^133^ANAL^136^ clades, highlighting viral adaptation driven by immune pressure. This recombinant strain has breakpoints at nucleotide positions 711 and 1629, covering the immunoreactive domain B of the capsid protein, linking recombination events to the epitope modification [63–65]. Similar patterns have been seen in Chinese PCV2b strains, where recombination led to epitope changes [66], indicating a pattern of immune‐driven viral evolution. Although infrequent, these events are epidemiologically important because they can generate mosaic genomes and distort phylogenetic relationships or dN/dS estimates if not excluded. Therefore, putative recombinant sequences were removed prior to phylogenetic, selection, and trait analyses in this study to ensure robust evolutionary inference.

Phylogenetic analysis of 2712 complete PCV2 genome sequences collected from Thailand and other Asian countries between 2007–2024 identified four major genotypes: PCV2a, PCV2b, PCV2d, and a small group of PCV2h. Among these, PCV2d was the most prevalent genotype. This trend agrees with observations from countries such as Vietnam, China, Korea, India, and Thailand, where PCV2d has also become the dominant genotype in recent years [10, 15, 16, 67–71]. However, China and some neighboring countries show higher PCV2 genetic diversity, including emerging genotypes not yet common in Thailand. PCV2d in China is also the dominant genotype, but there is greater diversity, with PCV2a, PCV2b and emerging genotypes like PCV2e and PCV2i detected in some regions [72, 73]. Vietnam, Malaysia, South Korea, and India also show PCV2d predominates, but PCV2b and intermediate genotypes (e.g., PCV2b‐IM1) are also found [17, 74]. The widespread presence of PCV2d across different regions, including its detection in Thai isolates from 2022 to 2024, suggests that virus evolution is likely driven by immune selection and vaccine pressure [61]. PCV2b, once dominant in the mid‐2000s, now appears to be declining. Significantly, some strains classified as recombinants, such as 19NPT29, were still grouped within the PCV2b [17], indicating that phylogenetic analysis alone may not reliably distinguish recombinant strains. This emphasizes the importance of incorporating recombination detection tools in genomic surveillance [66, 75]. Continued monitoring is essential to track the evolution and clinical relevance of these variants. Although PCV2a previously made up around 11% of circulating strains, recent studies show that it has become rare or undetectable in many Asian countries after 2020, suggesting a significant decline in its prevalence [18, 71, 76].

Selective pressure analysis is widely used to study how the PCV2 virus adapts, especially focusing on the capsid gene, which is the primary immunogenic protein. In this study, the selective pressure analysis reveals significant evolution, with five codons (63, 131, 134, 169, and 190) identified under positive selection. Codons 131 and 134 are located within immunoreactive domain B of the PCV2 capsid protein, a region targeted by neutralizing antibodies. Mutations at these sites can directly affect the antigenicity of the virus, leading to reduced binding by antibodies and facilitating immune escape [63–65]. In contrast, codons 63, 169, and 190 are not part of domain B but are found in other immunoreactive regions of the capsid protein [4, 77]. These findings highlight adaptive evolution driven by host‐pathogen interactions.

The phylodynamic analysis presented in this study supports the evidence of the viral evolutionary dynamics in Thailand from 2007 to 2024. The identification of three distinct PCV2d clades improves our understanding of the genetic diversity of PCV2 in Southeast Asia [10, 16, 18, 78]. The region spanning amino acids 133–136 in the capsid protein is recognized as an immunodominant domain [64, 65], and changes at this site may affect immune recognition and vaccine efficacy. In Thailand, a PCV2d variant with the amino acid motif ^133^HDAM^136^ has been recently reported [17]. This variant appears to have evolved from previous motifs ^133^ANAL^136^ and ^133^ATAL^136^. The ongoing presence of the ^133^ANAL^136^ clade since 2009, along with the decline of the ^133^ATAL^136^ clade by 2014, reflects the changing pattern of PCV2 evolution influenced by immune pressure. This finding is consistent with global trends indicating the predominance of the PCV2d genotype since 2010, likely driven by selective pressures from widespread vaccination and host immune responses [79, 80].

These phylodynamic patterns, together with evidence for multiple introductions, indicate that the coexistence of PCV2d clades (ATAL, ANAL, and HDAM) in Thailand likely reflects repeated importation events followed by local adaptation, rather than uninterrupted in‐country transmission. This interpretation provides a more accurate framework for understanding PCV2d molecular epidemiology within Southeast Asia.

This interpretation is further supported by our discrete trait analysis based on the Asia‐wide complete genome dataset, which revealed at least 12 independent introductions of PCV2 into Thailand between 2014 and 2021 (Supporting Information 4 and 5). Although this represents a minimum estimate within our regional sampling frame, it highlights repeated exchanges of PCV2 lineages between Thailand and neighboring Asian countries. Because the dataset was restricted to genomes reported from Asia, the actual number of introductions is likely higher, and some incursions may have originated from regions outside Asia (e.g., Europe or North America) that were not captured in this dataset. This pattern is consistent with previous reports describing regional livestock movement and transboundary pig trade networks in Southeast Asia [10, 61]. The coexistence of multiple PCV2d subclades in Thailand therefore likely reflects both importation events and subsequent local adaptation under immune or vaccine selection pressure, rather than isolated in‐country evolution.

The application of Bayesian skyride analysis represents an analytical approach to estimating viral population dynamics. The Bayesian skyride plot reveals a complex demographic history characterized by multiple phases of population expansion and contraction. The gradual increase in effective population size until 2007, followed by fluctuations through 2013–2019, and the subsequent decline continuing to the present, reflects the relationship between viral evolution, vaccination programs, and epidemiological conditions. This pattern demonstrates viral and host immune evolution, in which antigenic escape mutations lead to temporary increases in viral population size, followed by declines as the immune system adapts [9, 61]. The significant population contraction from 2013 to 2015 is concurrent with the period when widespread PCV2 vaccination was implemented in Thailand, supporting the possibility that vaccine‐induced immunity contributed to the decline in viral population. However, the subsequent population increase from 2015 to 2019 followed by resumed decline may indicate the emergence of vaccine escape variants, consistent with observations of novel antigenic variants in other region [7, 81, 82]. The amino acid changes of the capsid protein are associated with reduced vaccine efficacy and enhanced immune escape potential. The population dynamics revealed by the skyride plot analysis suggest that vaccination strategies should consider viral population change and potential for immune escape.

AlphaFold‐based structural predictions of the capsid proteins from PCV2 field strains, such as 374TH2023 and 10131TH2024 reveal critical surface‐exposed residues. Specifically, mutations identified at positions 133, 134, 136, and 169 were located on external surfaces of capsid proteins, which are located on neutralizing epitope targeted by antibodies [4, 64]. Structural modeling further reveals that these residues occupy flexible loops on the capsid exterior, allowing for antigenic drift [65]. T‐cell epitope analysis based on MHC binding affinity identified several candidate regions, with those showing high predicted binding scores selected for further analysis in this study. Most of the predicted T‐cell epitopes were located in variable regions of the capsid protein, suggesting that amino acid changes located in or adjacent to these sites may affect MHC binding. The epitope spanning positions 136–144 in the PCV2d ^133^HDAM^136^ clade exhibited strong predicted binding to MHC class I compared to other strains. In contrast, the epitope at positions 184–192 in the same clade showed lower predicted affinity for MHC class II. Mutations within these epitopes can alter their binding capacity, with some changes shifting the predicted affinity from strong to intermediate or weak, and vice versa, as reflected by IC50 values. For B‐cell epitope analysis, the ^133^HDAM^136^ and ^133^ANAL^136^ clades of PCV2d were selected due to their current prevalence in the field. The predicted linear B‐cell epitopes of these two strains shared closely similar positions, with minor differences in score due to sequence variation. Similarly, discontinuous epitope positions were largely conserved between the clades, but mutation affected their predicted antigenicity. Significantly, epitopes near the variable region at positions 133–136 demonstrated clade‐specific differences in both T‐ and B‐cell predictions, which may influence immune recognition and neutralization responses. The impact of such mutations was further supported by findings at position 169, where amino acid substitution altered epitope structure and predicted immunogenicity between clades. This finding is consistent with previous studies suggesting that amino acid mutations on the capsid surface of PCV2 may enhance the virus’s binding capacity to host immune cells [83]. The Thai recombinant strain 334TH2023 represents intragenotypic recombination between the ^133^HDAM^136^ and ^133^ANAL^136^ clades, introducing viral epitope and structural changes. This type of recombination may have contributed to the increase in viral population observed in the skyride plot between 2015 and 2019, a period during which recombinant variants likely escaped vaccine‐induced immunity [7, 81].

Thailand’s experience with PCV2 is similar to global trends, where the introduction and widespread use of vaccines, together with ongoing immune pressure, have driven genotype shifts and viral diversification [7, 61]. Before vaccination, PCV2a and PCV2b were the predominant genotypes. However, PCV2d became dominant around 2013–2014, when PCV2 vaccines began to be included in routine vaccination programs on most Thai pig farms around the early 2000 [10, 84]. Subsequent studies between 2019 and 2020 confirmed that PCV2d now represents over 80% of sequenced strains, with several new variants showing amino acid substitutions in immunoreactive regions of the capsid protein, suggesting immune‐driven adaptation [17]. Following vaccine introduction, selective pressure on capsid protein targeted by host immunity has increased, resulting in an accumulation of mutations within key epitopes and promoting partial immune escape [9, 81]. In addition to vaccine effects, as the result from this study, regional introductions and subsequent local evolution of new strains from neighboring countries have also increased the genetic diversity [10, 17]. These findings suggest that both vaccine pressure and regional viral movement have influenced the evolutionary of PCV2 in Thailand.

Effective prevention of PCV2 infection in Thailand requires an integrated approach combining vaccination, biosecurity, and continuous surveillance. Vaccination remains the primary control measure and both PCV2a‐based and bivalent (PCV2a/2b) vaccines have been shown to significantly reduce viremia, lesions, and transmission, even against the currently dominant PCV2d genotype [85, 86]. As PCV2d continues to evolve, multivalent or PCV2d‐based vaccines may provide better protection [87]. In addition, strengthening farm biosecurity and conducting regular genomic surveillance are important for detecting emerging variants and guiding future vaccine updates.

## 5. Conclusion

This study examined the complete genome and capsid gene of PCV2 strains circulating in Thailand and other parts of Asia from 2007 to 2024 to investigate viral evolution over time. Evidence of viral adaptation during this period was supported by results from phylogenetic and phylodynamic analyses, selective pressure and recombination assessments, Bayesian skyline plot–based demographic reconstructions, structural and epitope analyses of the capsid protein. Similar to recent studies, the majority of PCV2 strains circulating in Asia during this time were classified as genotypes PCV2d, PCV2b, and PCV2a, with a clear shift from PCV2a and PCV2b toward PCV2d [15, 16, 67, 69–71]. The viral population, as shown by the Bayesian skyline plot, displayed fluctuations likely influenced by factors such as vaccination and immune‐driven viral adaptation [9]. Recombination analysis further supported the ongoing evolution of PCV2. For instance, recombinant strains involving ^133^HDAM^136^ and ^133^ANAL^136^ clades demonstrated how intragenotypic recombination can give rise to variants with altered epitope profiles. To investigate specific genomic changes, selective pressure analysis was performed on the capsid gene. Several sites under positive selection were identified, including codons 133–136 and 169, which overlap with predicted epitope regions or residues associated with epitope binding. These changes likely affect host immune recognition. Additionally, discrete trait reconstruction using the Asia‐wide genome dataset revealed multiple independent introductions of PCV2 into Thailand between 2014 and 2021. Although the analysis was limited to Asian sequences, the observed genetic diversity in Thailand likely reflects both regional introductions and subsequent local evolution. Some introductions may have originated outside Asia, highlighting the need for broader global genomic surveillance to clarify international transmission routes.

In summary, this integrated analysis helps explain the evolution of PCV2 in Thailand and Asia and offers important insights into its molecular epidemiology. Ongoing genotype monitoring and antigenic surveillance are essential for the early detection of emerging variants. Structural analysis with conserved epitope mapping will support the development of improved vaccines with high effectiveness against circulating strains.

## Conflicts of Interest

The authors declare no conflict of interest.

## Funding

This research is funded by the National Research Council of Thailand (NRCT) (Grant Number N42A670624) and financial support provided by Zoetis Thailand for the conduct of this research.

## Supporting Information

Additional supporting information can be found online in the Supporting Information section.

## Supporting information


**Supporting Information 1** Data 1: Collection year of PCV2 strain and Genbank accession numbers used in the phylodynamic analysis.

**Supporting Information 2** Data 2: Detail of PCV2 strain and counties of origin used in the phylogenetic analysis.

**Supporting Information 3** Data 3: Summary of PCV2 recombinant strains, inferred parental sequences, breakpoint positions, and statistical support.

**Supporting Information 4** Data 4: JSON file of PCV2 interactive‐time‐scaled tree with country annotations.

**Supporting Information 5** Data 5: Details of PCV2 time‐scaled tree with country annotations.

**Supporting Information 6** Data 6: Structural models and Ramachandran plots of 374TH2023 and 10131TH2024 capsid protein.

## Data Availability

The data that supports the findings of this study are available in the supporting information of this article.
