# Supplementary material for: The Decade of Porcine Circovirus Type 2 (PCV2) in Thailand: Phylodynamic and Molecular Epidemiology
Source: Transbound Emerg Dis. 2025 Dec 6;2025:5565449. doi: 10.1155/tbed/5565449 (PMC12697814; doi:10.1155/tbed/5565449)
Supplement: Supplementary file 3 — Supporting Information 3 Data 3: Summary of PCV2 recombinant strains, inferred parental sequences, breakpoint positions, and statistical support. [file TBED-2025-5565449-s006.docx]

**Supplementary data 3**

Summary of PCV2 recombinant strains with inferred parental sequences, breakpoint positions, and statistical support.

| Recombinant | Minor | Major | Breakpoint position | | Lowest p-value |
| --- | --- | --- | --- | --- | --- |
|  |  |  | Begin | End |  |
| JF317582 | (KR704908) | JF317568 | 1136 | 1601 | 2E-19 |
| KC515002 | (OQ785638) | MZ474501 | 1089* | 1726 | 2.78E-09 |
| KJ680360 | MK421975 | MH465422 | 1848 | 1081 | 8.65E-82 |
| KP824712 | MK347404 | JF317587 | 1189* | 1570 | 1.39E-09 |
| KP824714 | MK426837 | (OM858856) | 514 | 1188 | 1.78E-12 |
| KP824715 | MT302482 | MK426836 | 1882 | 1038 | 8.31E-19 |
| KP824717 | (ON862530) | OQ785638 | 1037 | 1167* | 2.62E-08 |
| KP824719 | (JF827599) | JF317587 | 1232 | 1491 | 2.13E-12 |
|  | MK347404 | (MK426836) | 388 | 1040* | 2.02E-12 |
| KP824720 | OQ785638 | (KM360053) | 1862 | 1049 | 7.09E-19 |
| KP824722 | OQ785638 | (KY425815) | 1862 | 572 | 1.13E-26 |
|  | (KP824724) | KP824723 | 1794* | 1008 | 1.67E-23 |
| KP824723 | MK050982 | MK426836 | 420 | 1012 | 9.52E-12 |
| KP824724 | MZ667314 | (PP993528) | 422 | 1012 | 4.03E-19 |
| KR704903 | (OR537688) | OQ785638 | 1875 | 562 | 4.4E-17 |
|  | PP927788 | (HQ693092) | 789* | 1064 | 0.000000175 |
| KR704904 | MN170530 | JF317587 | 1212* | 1574* | 1.11E-08 |
| KR704910 | (KP081541) | KR704908 | 433 | 888 | 0.000000107 |
| KX960948 | (KR704903) | MN052983 | 1240 | 1640 | 1.86E-21 |
| MH094794 | OQ785638 | KR704908 | 1036* | 1862 | 3.69E-15 |
| MK426836 | (KP824714) | OR537652 | 452 | 1156 | 2.32E-09 |
| MK604507 | OQ785638 | KX929001 | 1502* | 1858 | 3.84E-14 |
| MW974888 | MK604511 | (OM858856) | 579 | 1008 | 5.72E-09 |
| PP927788 | KX929001 | KX929004 | 1741 | 788 | 2.24E-13 |
| PP993528 | (MF326355) | KP824719 | 442 | 979 | 3.64E-15 |
| PP993543 | KY810323 | KR704910 | 1858 | 888 | 5.99E-14 |
| GQ911590 | PP993548 | MK604482 | 1856 | 579 | 1.14E-09 |
| GU325757 | KP824712 | PP993543 | 1166* | 1530 | 7.83E-20 |
| KR704905 | MK426836 | (KX929001) | 1276 | 1693 | 3.91E-14 |
| KX929001 | (KM042405) | KP081541 | 1372 | 1596 | 0.00000152 |

(*) indicate positions with either overlapping recombination signals or slight uncertainties in the exact breakpoint location. The bracket represents cases where the recombinant was originally inferred to have an "Unknown" parent, but we revised the annotation by replacing “Unknown (Accession)” with the corresponding accession number alone in parentheses. The **lowest p-value** shown corresponds to the most significant value obtained across 7 recombination detection.
