# Supplementary material for: The Decade of Porcine Circovirus Type 2 (PCV2) in Thailand: Phylodynamic and Molecular Epidemiology
Source: Transbound Emerg Dis. 2025 Dec 6;2025:5565449. doi: 10.1155/tbed/5565449 (PMC12697814; doi:10.1155/tbed/5565449)

**Supplementary data 6** Structural model and Ramachandran plot of 374TH2023 and 10131TH2024 capsid proteins.

Structure model of 374TH2023 and 10131TH2024 (a, c) using PDB 3R0R as the template structure (gray), respectively. Ramachandran plots of 374TH2023 (b) and 10131TH2024 (d), respectively.


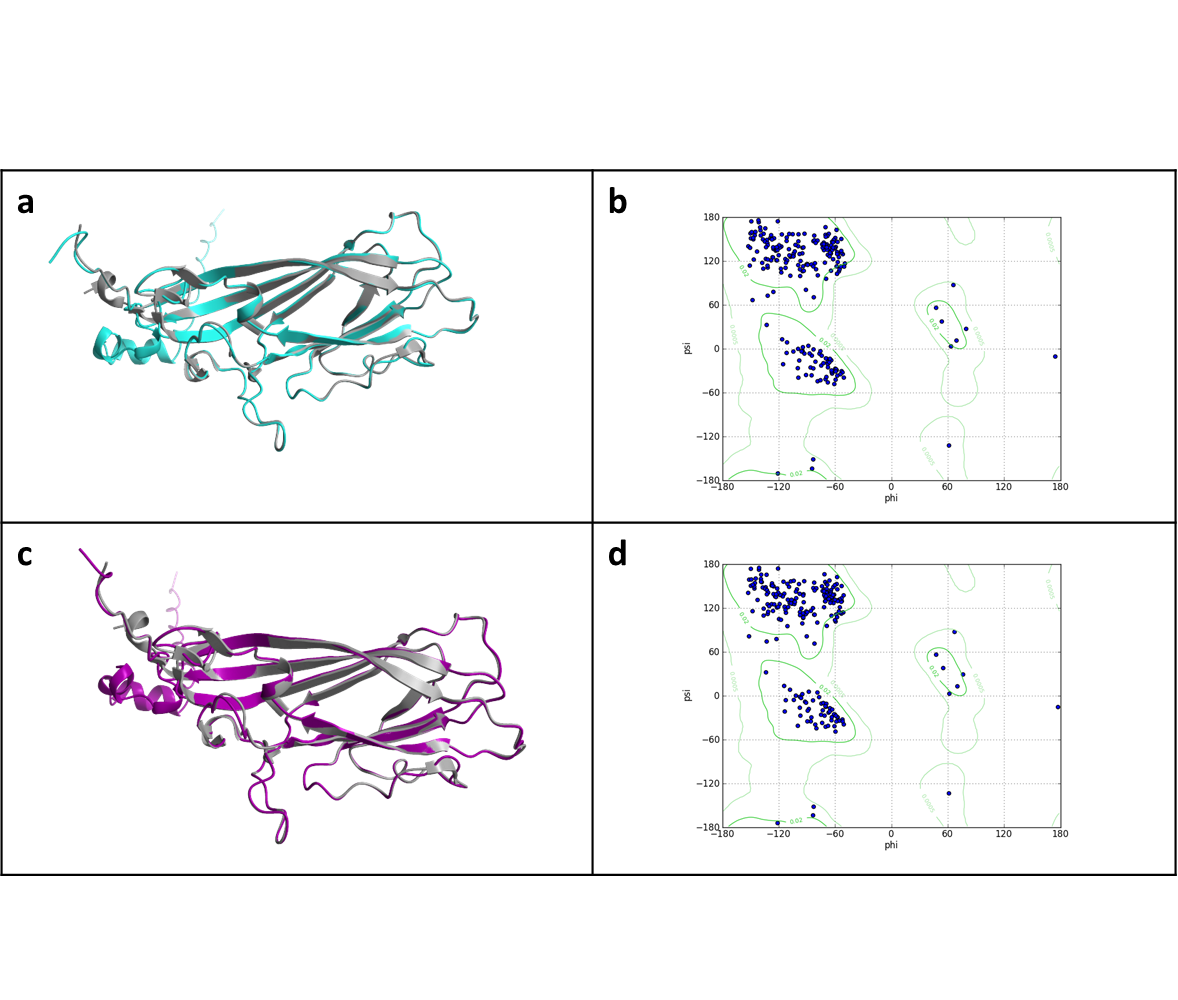

Supplement: Supplementary file 6 — Supporting Information 6 Data 6: Structural models and Ramachandran plots of 374TH2023 and 10131TH2024 capsid protein. [file TBED-2025-5565449-s003.docx]
